# Structural Features of Apicomplexan Pore-Forming Proteins and Their Roles in Parasite Cell Traversal and Egress

**DOI:** 10.3390/toxins9090265

**Published:** 2017-08-29

**Authors:** Alfredo J. Guerra, Vern B. Carruthers

**Affiliations:** Department of Microbiology and Immunology, University of Michigan, Ann Arbor, MI 48109-5620, USA; galfredo@umich.edu

**Keywords:** apicomplexan, parasite, pore-forming proteins, membrane disruption, cell traversal, egress, protein structure, regulation

## Abstract

Apicomplexan parasites cause diseases, including malaria and toxoplasmosis, in a range of hosts, including humans. These intracellular parasites utilize pore-forming proteins that disrupt host cell membranes to either traverse host cells while migrating through tissues or egress from the parasite-containing vacuole after replication. This review highlights recent insight gained from the newly available three-dimensional structures of several known or putative apicomplexan pore-forming proteins that contribute to cell traversal or egress. These new structural advances suggest that parasite pore-forming proteins use distinct mechanisms to disrupt host cell membranes at multiple steps in parasite life cycles. How proteolytic processing, secretion, environment, and the accessibility of lipid receptors regulate the membranolytic activities of such proteins is also discussed.

## 1. Introduction

Apicomplexans are obligate intracellular protozoan parasites that share a common set of apical secretory and cytoskeletal structures known as the apical complex. Notable members of the phylum Apicomplexa include *Cryptosporidium* spp., *Plasmodium* spp., and *Toxoplasma gondii*, which cause human cryptosporidiosis, malaria, and toxoplasmosis, respectively [1,2,3]. Other apicomplexans, such as *Eimeria* spp. and *Neospora caninum*, are important disease-causing agents in livestock, including poultry and cattle [4,5]. Apicomplexans replicate within a membrane-bound compartment, the parasitophorous vacuole (PV), in parasitized cells. Parasite escape from the PV after replication results in the cytolytic death of the host cell, causing the destruction of infected tissues and inducing inflammation, which together are the basis of the symptoms and disease. These parasites collectively exert a sizable toll on human and animal health, along with having a considerable economic effect.

*Plasmodium* and *Toxoplasma* are the most intensively studied apicomplexans because of their impact on human health, culturability, genetic tractability, and the availability of animal models for infection and disease [6]. These parasites have complex life cycles, involving an intermediate host for asexual replication and a definitive host wherein sexual replication culminates in a transmissible form. The infection of intermediate and definitive hosts requires substantial versatility by the parasite during its development in different types of host cells, along with an obligatory migration to distinct tissues to ensure efficient dissemination and transmission. As such, these parasites have evolved effective means for disrupting biological barriers, including membranes that confine or obstruct the parasites from progressing through their hosts.

Pore-Forming Proteins (PFPs) are often considered toxins because of their ability to create lesions in biological membranes, resulting in damage that compromises cell and tissue viability [7]. Many PFPs are synthesized in an inactive form and are secreted initially as soluble proteins that subsequently bind to target membranes and reconfigure or assemble into integral membrane pores [8]. Some PFPs function as portals to allow the translocation of other proteins and molecules, but the actions of many such proteins leads to the direct or indirect destruction of biological membranes [9,10]. PFPs can be binned into α- or β-PFPs, depending on whether membrane integration involves the insertion of α-helices or β-sheets.

**Figure 1:**
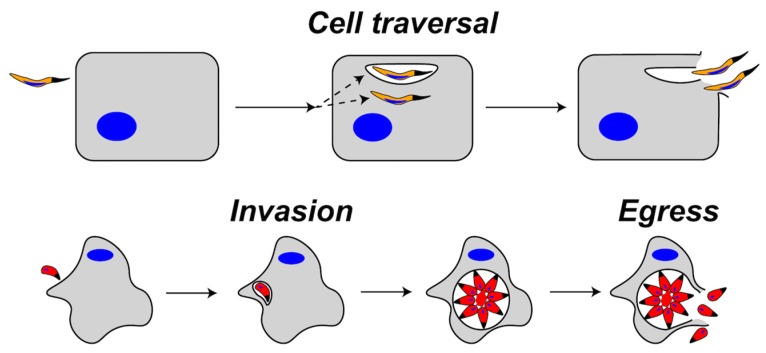
Representation of cell traversal (top) versus egress (bottom) by apicomplexans. During cell traversal, a sporozoite enters the host cell and is either contained in a transient vacuole or remains free in the cytosol. The parasite then exits the host cell by rupturing the transient vacuole and the host plasma membrane (HPM) or by traversing the HPM from the cytosol. During invasion, the parasite enters the cell and forms a parasitophorous vacuole (PV) where replication occurs. After replication, parasites egress from the PV and the host cell by rupturing the PV membrane and the HPM.

During apicomplexan infection, PFPs have been implicated mainly in two events termed cell traversal and egress (Figure 1). Both events involve the concerted action of PFPs and parasite gliding motility driven by a linear actomyosin motor system lying directly beneath the parasite plasma membrane. Cell traversal, which is crucial for migrating through tissues, involves the gliding-dependent penetration of the parasite into a target host cell [11,12]. During penetration, the parasite either forms a transient vacuole or it traverses the host plasma membrane (HPM) from the outside-in, rendering it free in the cytosol (Figure 1, top). Parasites residing in a transient vacuole exit in two steps: disruption of the transient vacuole (akin to the outside-in step) and inside-out traversal of the HPM [13]. A cytosolic parasite exits in a single step by directly rupturing the HPM from the inside-out. Egress, which is necessary to liberate the parasite after replication or development, occurs via the gliding- and PFP-dependent disruption of the PV and HPM (Figure 1, bottom) [14,15]. Cell traversal and egress are similar in that both involve parasite disruption of host-derived membrane barriers, but are distinguished by the timing of exit, which occurs within seconds for cell traversal and hours or days for egress.

Although the molecular and cellular underpinnings of cell traversal and egress remain poorly understood, recent crystallographic studies of known or putative apicomplexan PFPs have yielded important new insight into how such proteins contribute to cell traversal or egress. This review highlights the structural and functional advances for *Plasmodium* α-PFPs and discusses the role of membrane attack complex/perforin (MACPF) β-PFPs in *Plasmodium* and *Toxoplasma* infection biology.

## 2. Malaria Encodes Two PFPs That Are Unique among Apicomplexans

**Figure 2:**
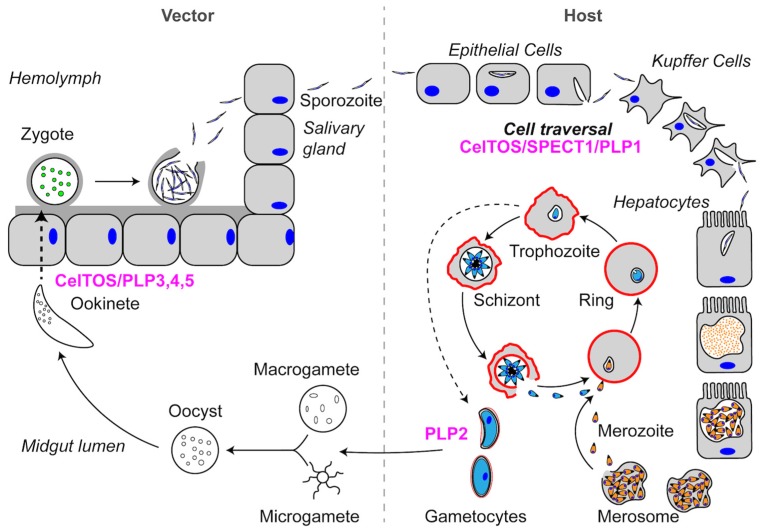
Schematic representation of the *Plasmodium* spp. life cycle. See text for descriptions of Pore-Forming Proteins (PFPs) and their roles in each step of the life cycle. PFPs are indicated in magenta.

To provide context for the stages relevant to PFP function, we briefly describe the life cycles of *Plasmodium* and *Toxoplasma* in this and subsequent sections. Further discussion of the life cycle can be found elsewhere [16,17]. The *Plasmodium* infection of a mammalian host begins when a sporozoite is injected into the host by an infected mosquito (Figure 2). Sporozoites are highly motile and migrate through the dermis to enter the blood stream and migrate to the liver. Once in the liver, sporozoites breach the liver sinusoid layer by traversing through Kupffer cells and hepatocytes until they create a productive PV. Sporozoites traverse cells by disrupting membranes to move out of the cell, a process first described in Kuppfer cells with the mouse malaria parasite *Plasmodium berghei* [18]. Similar to other *Plasmodium* spp., *P. berghei* encodes three proteins that are required for cell traversal: (1) Sporozoite microneme Protein Essential for Cell Traversal 1 (SPECT1); (2) Sporozoite microneme Protein Essential for Cell Traversal 2 (SPECT2; also known as Perforin-Like Protein 1 (PLP1)); and (3) Cell-Traversal protein for Ookinetes and Sporozoites (CelTOS) (Table 1). In addition to functioning in sporozoite cell traversal, CelTOS, as the name implies, is also required for ookinete cell traversal in the mosquito stage. *Plasmodium* parasites additionally encode four other Perforin-Like Proteins (PLP2-5), some of which also play a role in ookinete cell traversal. All of these proteins are released from the parasite micronemes, which are calcium-responsive apical secretory organelles that are discharged during cell traversal, egress, and invasion [19]. In the following sections, we will discuss SPECT1 and CelTOS, since these two proteins are unique to *Plasmodium* parasites. PLP proteins will be discussed more extensively in later sections.

### 2.1. SPECT1: A Unique Plasmodium Protein for Cell Traversal

#### 2.1.1. SPECT1 and Cell Traversal

*P. berghei* sporozoites lacking SPECT1 (*Pb∆spect1*) have wild-type-like gliding motility and hepatocyte infectivity, but are unable to traverse sinusoidal layer cells and thus incapable of accessing hepatocytes in the parenchyma of the liver [20]. Treatment with liposomal clondronate, which depletes Kupffer cells, restored normal levels of infection presumably due to the ability of the parasites to glide through the gaps formed by Kupffer cell depletion. *Pb∆spect1* parasites are 5- to 10-fold less infective than wild-type parasites when injected subcutaneously. Additionally, most *Pb∆spect1* sporozoites are trapped in mouse dermis after deposition from a mosquito bite, despite having normal gliding motility in three-dimensional (3D) matrices [12]. Recent work has also shown that *Plasmodium falciparum* SPECT1 (*Pf*SPECT1) contributes to *P. falciparum* infection of mice with humanized liver tissue [21]. SPECT1 is highly conserved among *Plasmodium* species (~40% sequence identity), consistent with sharing a common structure and function. Indeed, BLAST searches querying apicomplexan genomes with SPECT1 only return matches within the *Plasmodium* genus.

#### 2.1.2. *P. berghei* SPECT1 Crystal Structure: A Common Structural Fold Associated with Membrane Proteins

**Figure 3:**
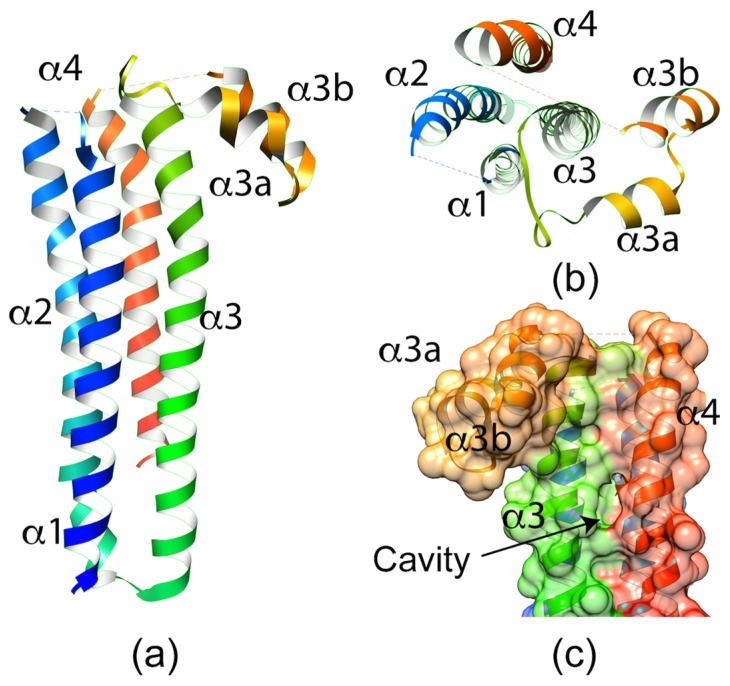
(**a**) Side view of the crystal structure of *Plasmodium berghei* SPECT1. The structure shows a four-helix bundle with a hook-like element between α3 and α4. (**b**) Top view of the SPECT1 crystal structure. The dashed lines between α1–α2 and α3b–α4 represent loops lacking electron density. (**c**) Surface representation of the back of the SPECT1 crystal structure showing the cavity formed between α3 and α4.

Recent solving of the 2.7 Å crystal structure of *Pb*SPECT1 provided some intriguing, albeit cryptic, potential clues to its function in cell traversal [22]. The structure shows a nearly parallel four α-helix bundle with a “hook” feature on one end (Figure 3). The four-helix bundle is a fairly common structural fold found in a subset of transmembrane proteins. Accordingly, a Dali Lite server [23] search with the *Pb*SPECT1 structure yields similarity to a THATCH domain of Huntingtin-interacting protein 1 (HIP1R THATCH, PDB code 1R0D) [24], a coiled-coil domain of a Potato Virus X Resistance Protein (RxCC, PDB code 4m70) [25], a transmembrane domain of a eukaryotic V-type ATPase (PDB code 5VOZ) [26], and a yeast t-SNARE protein (Sso2, PDB code 5M4Y) [27]. The last two of these proteins are either transmembrane domains or have membrane-binding activity. However, no transmembrane helices are predicted in the *Pb*SPECT1 sequence, and it remains unclear if or how SPECT1 interacts with membranes during *Plasmodium* cell traversal. One enticing feature of the crystal structure is a rather deep pocket flanked by the α-3 and α-4 helices (Figure 3c). This cavity is lined primarily with hydrophobic residues that are conserved in other SPECT1 homologs, and could be a binding site for cholesterol or lipids. However, another plausible interpretation of this cavity and the parallel α-helices is that they indicate a measure of instability in the crystallized structure. This could be telling of potential conformational changes that may occur in SPECT1. Interestingly, the structure of *Pb*SPECT1 also bears a striking similarity to the N-terminal portion of a mammalian protein termed Izumo1, which also consists of a nearly parallel four α-helix bundle with a hook feature [28,29]. This so-called Izumo domain is crucial to the function of Izumo1 in the fusion of mammalian sperm with an egg. SPECT1 and the Izumo domain share solvent-exposed bulk hydrophobic amino acids speculated to interface with other proteins. Although additional studies are necessary to understand how SPECT1 and the Izumo domain confer membrane traversal or fusion, these crystallographic studies provide an important basis for future work identifying associated proteins and mechanisms underlying membrane interaction.

### 2.2. CelTOS: A Conserved Protein in the Aconoidasida Is Required for Cell Traversal in Both the Mammalian Host and Vector

#### 2.2.1. CelTOS Dictates Cell Traversal by Sporozoites and Ookinetes

CelTOS is unique to the Aconoidasida class of apicomplexan parasites, which includes *Plasmodium* spp. and the tick borne parasites *Babesia* spp. and *Theleria* spp. Genetic studies in *Plasmodium* established the essentiality of CelTOS for efficient traversal in both the mammalian host (sporozoite) and mosquito vector (ookinete). Indeed, CelTOS is only expressed by ookinetes and salivary gland sporozoites, but not by immature sporozoites in the oocyst [30]. Whereas ∆*celtos* parasites show normal ookinete development in vivo or in vitro, they exhibit a marked defect in migration through midgut epithelial cells, resulting in 200-fold reduction in subsequent oocysts. Similarly, ∆*celtos* sporozoites show normal microneme secretion, but have reduced infectivity based on exhibiting a longer prepatent period upon the infection of mice. This phenotype is reversed when sporozoites lacking CelTOS are inoculated in Kupffer cell-depleted rats. Finally, despite having a similar ability to form PVs in a HEPG2 cell line, ∆*celtos* sporozoites have much lower cell wounding activity. Taken together, these experiments highlight the importance of CelTOS in both ookinete and sporozoite cell traversal, rendering this protein a potential vaccine target for preventing liver infection [31,32].

#### 2.2.2. CelTOS Is a PFP with a Preference for Phosphatidic Acid

**Figure 4:**
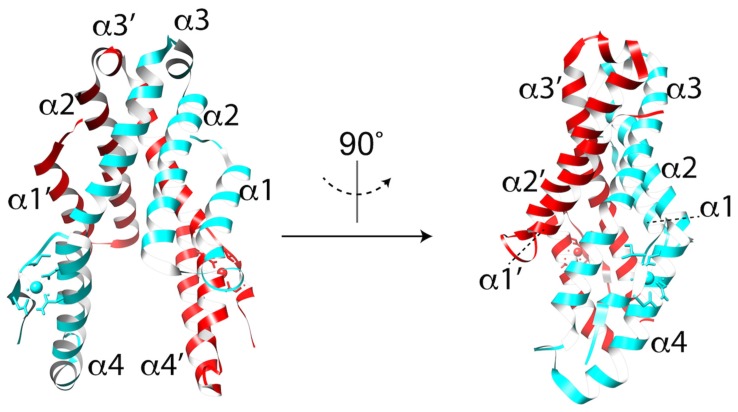
Crystal structure of the CelTOS dimer in ribbon representation with each monomer colored in cyan and red. The N-terminal subunit is defined by helices α1 and α2. The C-terminal subunit is defined by helices α3 and α4.

Despite its importance as a cell traversal protein and being a conserved protein within a subset of apicomplexan parasites, the mechanism by which CelTOS aides in cell traversal has remained elusive largely due to a lack of similarity to other proteins of known function. Recent solving of the *Plasmodium vivax* CelTOS (*Pv*CelTOS) 3.0 Å crystal structure revealed a highly α-helical dimer resembling a tuning fork (Figure 4) [33]. Although the overall structure lacks similarity to canonical pore-forming proteins, the N-terminal and C-terminal subunits bear some resemblance to the membrane disruptive viral and bacterial proteins HIV-gp41, Hendravirus fusion protein, Nipahvirus fusion protein, and *Mycobacterium bovis* ESAT-6 [33]. Additionally, both *Pf*CelTOS and *Pv*CelTOS bind phosphatidic acid (PA) in a spotted lipid array, and at nanomolar protein concentrations can disrupt liposomes containing PA [33]. Liposomes containing phosphatidylserine or phosphatidylcholine required micromolar concentrations of CelTOS. If PA is the physiologic receptor for CelTOS, the known localization of PA to the inner leaflet of the HPM implies a role for CelTOS in the inside-out disruption of the HPM for sporozoite exit. Finally, transmission electron microscopy of negative stained liposomes with and without CelTOS show the formation of CelTOS-dependent ~50 nm pores [33]. These studies provide important new insight into how CelTOS facilitates parasite cell traversal and lay a foundation for future work identifying the structural rearrangements associated with pore formation.

## 3. Apicomplexans Share a Family of MACPF PFPs that Function at Multiple Steps in Their Life Cycles

### 3.1. MACPF/CDC Proteins Have a Variable C-Terminal Domain

**Figure 5:**
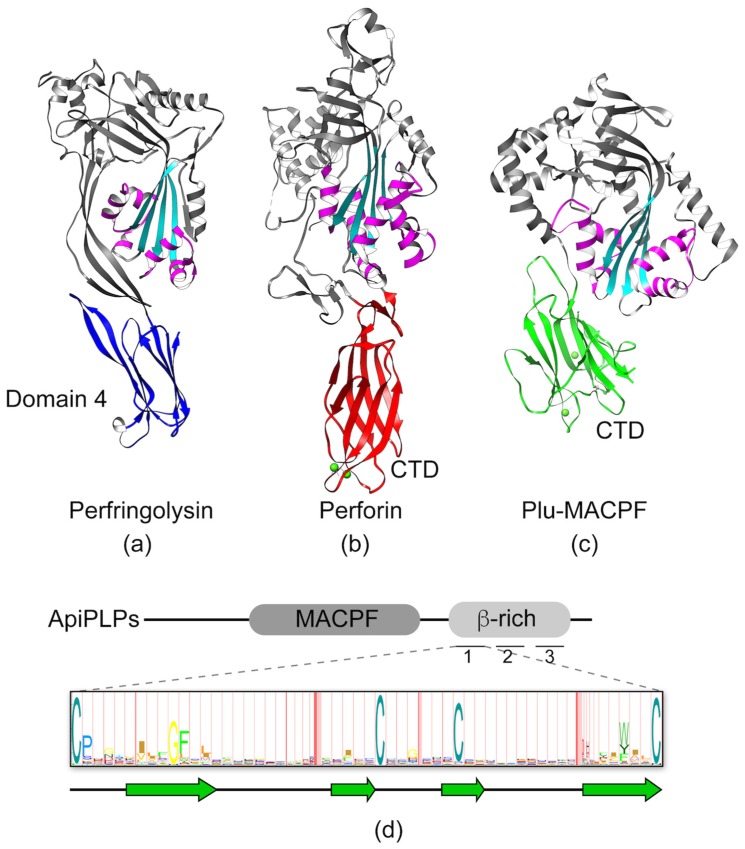
Representative structures of the membrane attack complex/perforin (MACPF)/ cholesterol-dependent cytolysins (CDC) family of proteins. The conserved MACPF/CDC domain is shown in cyan and two conserved helical clusters (CH1/CH2) are colored in magenta. (**a**) Structure of Perfingolysin (PDB code 1PFO) with domain 4 highlighted in blue. (**b**) Structure of Perforin 1 (PDB code 3NSJ) with the C-terminal domain (CTD) colored in red. Calcium ions in the CTD are shown as green spheres. (**c**) Structure of Plu-MACPF (PDB code 2QP2) with the C-terminal domain colored in green. Calcium ions in the CTD are shown as green spheres. (**d**) Diagram of the domain organization of a generic ApiPLP illustrating the MACPF and β-rich domains. The domain is made up of three direct repeats of ~60 amino acids, each containing conserved cysteine and hydrophobic amino acids illustrated in the Hidden Markov Model (HMM) logo created from 51 individual repeats representing 22 ApiPLPs (*Tg*PLP1-2, *Neospora caninum Nc*PLP1-3, *Pf*PLP1-5, *Babesia bovis Bb*PLP1-6; *Theileria annulata Ta*PLP1-6). The degree of conservation at each position is proportional to the size of the single letter code indicating the residue. Approximate positions of predicted β-sheets are depicted below the HMM logo.

MACPF proteins are members of a large and diverse family of pore-forming proteins found in virtually all kingdoms of life, including apicomplexans [19,34,35]. The pore-forming MACPF domain, which has structural similarity to domains 1 and 3 of cholesterol-dependent cytolysins (CDCs), is defined by a central four-stranded antiparallel β-sheet (Figure 5a, cyan) and two clusters of α-helices termed CH1 and CH2 (Figure 5a, magenta). The first step in pore formation involves membrane recognition and binding. In CDCs, this occurs via the conserved domain 4 (Figure 5a, blue), which is comprised of an immunoglobulin-like fold that recognizes cholesterol and initiates binding to the membrane. MACPF proteins, on the other hand, have significant variation in the C-terminal domain (CTD), and the membrane receptors for these proteins remain unknown. The variability in CTD structures becomes evident when comparing the crystal structures for Perforin 1 (Figure 5b; C2 domain fold) and Plu-MACPF (Figure 5c; β-prism fold). Apicomplexan MACPF proteins, termed Perforin-like proteins (ApiPLPs), share the conserved MACPF domain and a downstream β-rich domain (Figure 5d). The β-rich domain is similar to the CTDs of CDC and other MACPF proteins in that it is predicted to consist mainly of β-pleated sheets. However, unlike other CTDs, the β-rich domain is comprised of three direct repeats, each containing four highly conserved cysteine residues that are presumed to form disulfide linkages. Several hydrophobic residues are also partially conserved. The extent to which the repeats form a single globular domain or three tandemly linked mini-domains remains unknown. The signature repeat is conserved among the β-rich domains of ApiPLPs, but otherwise the domain sequence has no homology to other proteins. Although an in silico molecular modeling study [36] suggested that the β-rich domain of *Plasmodium* PLPs resembles a C2 domain fold, C2 domains do not typically contain repeat elements and the C2 signature sequence (Pfam ID number PF00168) bears no resemblance to the β-rich motif. Thus, it remains to be determined whether the ApiPLP β-rich domain is structurally related to other CTDs of MACPF proteins such as the C2-domain of perforin. Regardless, after binding of a MACPF protein via its CTD to the target membrane, the protein oligomerizes into ring- or arc-shaped complexes. These so-called pre-pore complexes then undergo a dramatic structural rearrangement of the MACPF domain, wherein the CH1 and CH2 helices unfurl to become extensions of the central β-sheets, thereby knifing into the target membrane to form a pore as a super β-barrel. *T. gondii* PLP1 (*Tg*PLP1) and *P. falciparum* PLP1 (*Pf*PLP1) were shown to bind membranes, oligomerize, and have pore-forming activity [37,38,39,40]; thus, it appears that ApiPLPs share the basic mechanism of pore formation with other MACPF proteins.

### 3.2. Plasmodium MACPF Proteins Are Important for All Stages of the Parasite Life Cycle

#### 3.2.1. Role of *Plasmodium* PLP1 in Cell Traversal

As mentioned above, during the infection of the mammalian host *Plasmodium*, sporozoites must traverse a variety of cell types before developing into a non-motile, replicating exo-erythrocytic form (EEF) inside a hepatocyte (Figure 2). Cell traversal of dermal and liver sinusoidal layer cells is dependent on both SPECT1 and PLP1. Sequence alignments show the similarity of *Pf*PLP1 to other MACPF proteins, including a signature motif (Y/W-G-T/S-H-F/Y-X6-G-G) as well as helical clusters, CH1 and CH2. Previous work reported that *Pf*PLP1 was expressed in blood stages and mediates the calcium-dependent egress of merozoites from erythrocytes [39]. However, studies with *P. falciparum* parasites expressing a triple HA9 epitope endogenously tagged PLP1 failed to detect expression of *Pf*PLP1 during the blood stage [41]. Additionally, a deletion strain of *Pf*PLP1 (*Pf∆plp1*) showed normal asexual growth when cultured in erythrocytes in vitro [41]. *Pf∆plp1* parasites also are not defective in a number of gametocytes, midgut oocysts, or salivary gland sporozoites. *Pf∆plp1* sporozoites are, however, deficient in the cell traversal of both hepatocytes and human monocyte-derived macrophages. This defect appears to be confined to cell traversal, since *Pf∆plp1* parasites showed normal invasion of hepatocytes and EEF development. Finally, the intravenous inoculation of *Pf∆plp1* sporozoites into mice with humanized liver tissue failed to establish infection, whereas wild-type parasites readily infect hepatocytes in this same system. These observations, which are consistent with earlier studies of *P. berghei* parasites lacking *Pb*PLP1 [12,42,43], suggest a principal role for *Plasmodium* PLP1 in cell traversal during sporozoite migration from the dermis to the blood stream and movement across the liver sinusoidal layer into parenchymal hepatocytes.

Initial work describing hepatocyte cell traversal by rodent *Plasmodium yoelii* sporozoites suggested a model in which the parasite crosses the HPM from the outside-in and it temporarily occupies the host cytosol before exiting the cell via an inside-out rupture of the HPM [11]. This model was based mainly on the observation by electron microscopy of sporozoites lacking a vacuolar membrane. Other work with *P. berghei* or *P. yoelii* sporozoites and Kupffer cells, however, observed sporozoites within a vacuole, putatively during the cell traversal of such cells [18,44]. Nevertheless, these hepatocyte and Kupffer cells studies did not directly observe the fate of the HPM or vacuole membrane during cell traversal. More recent work carefully examining temporal steps in cell traversal concluded that the majority of cell-traversing *P. yoelii* sporozoites occupy a transient vacuole in hepatocytes, and that escape from the transient vacuole requires the expression of *P. yoelii* PLP1 (*Py*PLP1) [13]. Failure to escape resulted in the fusion of hepatocyte lysosomes with the vacuole, leading to sporozoite death. These findings suggest an intriguing new facet of cell traversal involving a transient vacuole. Additional work is needed to define the extent to which transient vacuoles exist in vivo or are used by other *Plasmodium spp*.

#### 3.2.2. *Plasmodium* PLP2 Functions in Gametocyte Egress

After exiting the liver stage, *Plasmodium* parasites infect erythrocytes and undergo a second round of asexual reproduction in the symptomatic erythrocytic stage (Figure 2). Generally, the erythrocytic cycle is separated into three developmental stages: ring, trophozoite, and schizont. However, a small percentage of trophozoites differentiate into gametocytes within erythrocytes, from which they must egress for mating in the mosquito midgut. Gametocyte egress is dependent on a second perforin-like protein (PLP2) expressed in male gametocytes [45]. A strain of *P. berghei* lacking PLP2 shows normal development of gametocytes in the blood stage, but the gametocytes are unable to egress and have aberrant exflagellation. This phenotype is overcome by treatment with saponin or the pore-forming toxin equinatoxin II prior to when erythrocytic membrane rupture would normally occur. Mutant *P. falciparum* parasites lacking PLP2 also showed normal egress during the intraerythrocytic cycle, but aberrant egress of gametocytes that is rescued by equinatoxin II treatment. These studies suggest that *Plasmodium* does not use MACPF proteins for cell traversal exclusively, but also deploys them for egress from infected cells in a manner akin to *T. gondii* (see below).

#### 3.2.3. *Plasmodium* PLP3,4,5 and Cell Traversal in the Mosquito Midgut

Following the ingestion of a blood meal from an infected host, male and female *Plasmodium* gametocytes mate to generate a motile zygote known as an ookinete. Ookinetes must traverse the midgut epithelium to avoid mosquito immune defenses and access the basolateral membrane for further development into oocyts. This step is a major bottleneck in the lifecycle, which is likely why the parasite has evolved four individual PFPs to mediate the traversal of midgut epithelium. One of these, CelTOS, is described above. The other three PFPs are MACPF proteins termed PLP3 (also known as MAOP), PLP4, and PLP5.

*P. berghei* PLP3 (*Pb*PLP3) is expressed in the ookinete stage, where it localizes to micronemes. Parasites lacking *Pb*PLP3 (*Pb∆plp3*) show a striking lack of infectivity in mosquitoes and are incapable of traversing epithelial cells. *Pb*PLP3-deficient ookinetes can attach to the apical surface of the midgut epithelium, but are unable to disrupt the HPM of epithelial cells [46].

*P. falciparum* ookinetes show a large increase in PLP4 (*Pf*PLP4) transcript levels compared to blood or gametocyte stages. *Pf*PLP4 deficient parasites have normal blood stage replication, gametocytogenesis, and exflagellation, suggesting that *Pf*PLP4 is not important during these life cycle events. However, feeding mosquitoes with the mature gametocytes of *Pf∆plp4* parasites results in a significant reduction of oocysts when compared to a wild-type control, consistent with an inability to traverse midgut epithelial cells.

Similarly, a *P. berghei plp5* deletion strain (*Pb∆plp5*) can bind to the apical side of the midgut epithelium, but is unable to traverse epithelial cells. However, this lack of infectivity is not absolute, since a small number of oocysts and salivary gland sporozoites were detected in infected mosquitoes. Interestingly, the direct injection of *Pb∆plp5* ookinetes into the hemocoel, which effectively bypasses traversal of the midgut epithelium [47], restores the numbers of oocysts and salivary gland sporozoites, pinpointing the role of *Pb*PLP5 in the traversal of the midgut epithelium.

It is interesting to note that, despite the presence of four PFPs (CeLTOS, PLP3,4,5) during ookinete midgut epithelium invasion, there appears to be little functional overlap in this process. The possibility that pore formation by PLP3,4,5 might require heteromultimerization was proposed as a potential explanation for the apparent lack of functional redundancy [34]. These proteins might also act at different steps, namely the outside-in and inside-out traversal of the epithelial cell HPM, as suggested previously [48]. It should be noted that pore formation by PLP3,4,5 has not been established, but rather is inferred based on membership in the MACPF family. Further studies into the individual roles of CelTOS and PLP3, 4, and 5 are necessary to understand the mechanism by which malaria ookinetes traverse the midgut epithelium.

### 3.3. Toxoplasma MACPF Proteins

#### 3.3.1. *Toxoplasma* Infection of Its Hosts

**Figure 6:**
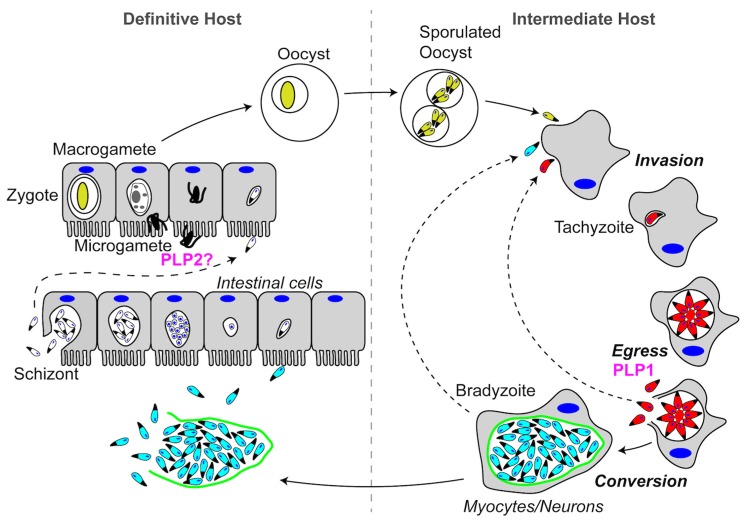
Schematic representation of the *Toxoplasma gondii* life cycle. PFPs that are important to the life cycle are labeled in magenta.

The *Toxoplasma gondii* life cycle is simpler than the *Plasmodium* life cycle, but still involves the infection of two different hosts. Again, we will only briefly discuss the life cycle to give context to our discussion of PFPs. The *Toxoplasma* life cycle can be generally divided into feline and non-feline infections (Figure 6). Sexual reproduction only occurs during the infection of felines, the definitive host. *T. gondii* is capable of infecting and replicating asexually within virtually all warm-blooded vertebrates, thus it is highly promiscuous for intermediate hosts. Infection begins with the consumption of a tissue cyst or a sporulated oocyst. During invasion, parasites form a PV wherein tachyzoites replicate asexually to form vacuoles containing 16 or more parasites. Tachyzoites then egress from the cell and invade neighboring host cells. This sequence of invasion, asexual reproduction, and egress constitutes the lytic cycle of acute infection. Following the acute infection, tachyzoites undergo differentiation into slow-growing bradyzoites, which develop into intracellular tissue cysts during chronic infection. If the tissue cyst is consumed by a non-feline intermediate host, the asexual reproductive cycle begins again. If a felid consumes the tissue cyst, the parasites undergo schizogony and sexual differentiation to form oocysts that are shed in the feces. In the following section, we will describe the role that *T. gondii* PLP1 (*Tg*PLP1) plays during egress and postulate a putative role for *T. gondii* PLP2 (*Tg*PLP2) during the sexual stage.

#### 3.3.2. *Toxoplasma* PLP1 & PLP2

The *Toxoplasma gondii* genome encodes two PLP proteins, *Tg*PLP1 and *Tg*PLP2. A sequence analysis and homology modeling confirm that both proteins have the conserved features of a MACPF protein, including the signature sequence motif and the CH1 and CH2 regions in the pore-forming domain [15]. Interestingly, similar to the CTDs of MACPF family proteins, the C-terminal domains of *Tg*PLP1 and *Tg*PLP2 are predicted to be rich in β-pleated sheets, but they bear no sequence homology with other MACPF CTDs, as noted above. The *Tg*PLP1 CTD is required for pore-forming activity [37]. *Tg*PLP1 is secreted from micronemes in a calcium-dependent manner, and facilitates parasite egress by disrupting the PV membrane [15]. Video microscopy experiments showed that *Tg∆plp1* parasites became motile within the PV after stimulating calcium signaling, but they show a substantial delay in crossing the PV membrane and exiting the host cell. *Tg∆plp1* parasites also have a defect in spontaneous egress, based on observing large clusters of parasites trapped in “spherical structures” in host cells during routine culture. Further examination by electron microscopy revealed that these spheres were contained by both the vacuolar membrane as well as the host plasma membrane. Interestingly, mice infected with high doses of *Tg*PLP1-deficient parasites survive the infection, implicating *Tg*PLP1 as a key virulence factor [15]. In addition to the central MACPF domain and a β-rich CTD, *Tg*PLP1 has an N-terminal domain that has also been shown to have membrane-binding activity [37]. Although this domain is dispensable for pore formation and virulence, parasites expressing *Tg*PLP1 lacking the N-terminal domain egress less efficiently from host cells than those expressing the complete *Tg*PLP1, indicating a contributing but non-essential role in PLP1 function.

Transcriptional profiling (Toxodb.org) and epitope tagging [15] experiments suggest that *Tg*PLP2 is not expressed during acute asexual infection, and instead is produced during sexual development in the intestinal system of felids when *Tg*PLP1 is transcriptionally silent. As such, *Tg*PLP2 might play a role in gametocyte egress analogous to *Plasmodium* PLP2.

## 4. Regulation of Apicomplexan PFPs

Intracellular PFP activity is often limited in eukaryotic cells by producing the protein in a pro-form and by localization to a secretory organelle. For example, perforin 1 activity in cytotoxic T cells is controlled during intracellular transport by a C-terminal propeptide including a terminal tryptophan, which mediates rapid export from the ER, but is not required for cytolysis [49,50]. Deletion of the propeptide or mutation of Trp led to perforin 1 accumulation in the ER and cytotoxicity. *Tg*PLP1 and *Tg*PLP2 are likewise produced as pro-proteins with an N-terminal signal anchor sequence that is sufficient for *Tg*PLP1 targeting to micronemes [36]. Although the subcellular location at which the *Tg*PLP1 signal anchor propeptide is cleaved is not known, tethering *Tg*PLP1 to the luminal membrane by the N-terminal signal anchor may prevent autolysis during trafficking by preventing the C-terminal domain from binding the membrane. However, the mechanisms that suppress activity once maturation has occurred and the protein is stored in the micronemes remain to be determined. *Plasmodium* PLPs lack a signal anchor sequence and instead possess a signal sequence for translation into the ER lumen. It remains unclear if *Plasmodium* PLPs contain autoinhibitory sequences that suppress cytolytic activity during trafficking to and storage within the micronemes.

Storage within micronemes permits the regulated release of apicomplexan PFPs precisely when and where they are needed to disrupt biological membranes during cell traversal and egress. Calcium signaling within the parasite triggers the microneme release of PFPs along with activating parasite motility, which together deliver the cytolytic activity and mechanical force necessary for cell traversal and egress. How the parasite remains seemingly immune to the actions of its own PFPs is unknown, but prospectively could involve inhibitory proteins on the parasite surface or the lack of specific lipid or protein receptors.

Extracellular *Toxoplasma* tachyzoites secrete *Tg*PLP1 and other microneme proteins in response to a decrease in pH of the medium via activation of calcium signaling in the parasite [15,37]. In addition to stimulating the release of *Tg*PLP1, low pH also promotes the cytolytic activity of *Tg*PLP1 by enhancing membrane binding via its CTD [38]. The combined actions of increased release and enhanced activity in low pH medium rendered tachyzoites capable of wounding host cells in a *Tg*PLP1-dependent manner. Such activity is not normally seen in tachyzoites, which typically invade into a PV without damaging host cells. Additional work provided evidence on a population scale that the PV of intracellular dividing tachyzoites acidifies near the time of egress. Also, treatments that antagonize acidification impaired parasite egress from host cells upon stimulation of calcium signaling. Interestingly, the pretreatment of hepatocytes with bafilomycin A, which blocks V-type ATPase-dependent proton transport, trapped wild-type *Plasmodium* sporozoites in transient vacuoles [13]. Since escape from transient vacuoles is dependent upon the expression of *Plasmodium* PLP1, the findings imply that the acidification of the transient vacuole, possibly by the fusion of host lysosomes, promotes PLP1 secretion and/or activity. Together, these studies suggest a working model in which the acidification of *Toxoplasma*- and *Plasmodium*-containing vacuoles promotes PLP1-dependent membranolytic activity for egress or cell traversal, respectively. However, it should be noted that the pH of individual PVs or transient vacuoles prior to egress or escape has not been measured directly. Thus, additional work is needed to validate vacuole acidification and identify the underlying mechanism(s).

In addition to regulation by autoinhibitory sequences, storage in micronemes, and environmental pH, a fourth level of regulation is likely conferred by receptor availability, exemplified by CelTOS [33]. As mentioned above, PA is typically enriched in the inner leaflet of the HPM, thereby supporting a role for CelTOS in inside-out disruption of the HPM during cell traversal. In this scenario, CelTOS would not act optimally on an outside-in rupture of the HPM or transient vacuole, which instead is attributed to *Plasmodium* PLP1 activity. By extension, other apicomplexan PLPs may recognize leaflet-specific receptors that dictate the directionality of their actions. In this way, receptor accessibility could also contribute to the directionality of *Tg*PLP1’s role in promoting tachyzoite egress from host cells without compromising the formation of an intact PV during subsequent invasion.

## 5. Perspectives

In this review, we have summarized recent insight gained from structural studies of apicomplexan PFPs and their roles in infection. Current evidence suggests that PFPs are important factors throughout the *Plasmodium* and *Toxoplasma* life cycles, but several important questions remain unanswered. First, despite the evidence that *Pf*SPECT1 is required for cell traversal, its activity as a PFP remains uncertain. Even with the crystal structure in hand, the mechanism by which *Pf*SPECT1 potentially forms pores remains unknown. In the same vein, the crystal structure of *Pf*CelTOS bears some resemblance to PFPs from other organisms, but it is unclear if or how *Pf*CelTOS recognizes PA during cell traversal. Furthermore, if PA is the cognate receptor for *Pf*CelTOS, there is a strong implication of protein activity in the latter portions of cell traversal, as PA is primarily an inner leaflet phospholipid; however, further work is necessary to confirm this hypothesis. Additionally, although there is evidence that *Pf*CelTOS forms pores, the extent of the structural rearrangements associated with pore formation remains obscure. High-resolution cryoelectron microscopy of *Pf*CelTOS has the potential to identify such structural rearrangements.

Despite our current knowledge of the structures of MACPF/CDC proteins in other organisms, we currently lack any empirical information on the structures of *P. falciparum* or *T. gondii* PLPs. This is particularly important considering the unique features of MACPF CTDs. The CTD is a key anchor point in the mechanism for pore formation. Solving the structures of the ApiPLPs will allow further understanding of how this set of proteins bind membranes prior to pore formation. Structural studies of ApiPLPs should also aid in understanding the basis for pH-dependent PLP activity. Further studies are also necessary to identify the receptors for ApiPLPs, since these may shed light on the directionality, i.e., inside-out or outside-in, of membrane disruption by these proteins. Finally, it is likely that there are other factors that influence parasite egress. Generally, the pores formed by the proteins described herein (50–200 Å or 5–20 nm) are much smaller than the girth of individual parasites (1000–2000 nm). While it is plausible that pore insertion destabilizes membranes sufficiently to allow traversal via gliding motility, PFPs might also work in collaboration with other membrane active proteins that enlarge the pores or further weaken the membrane integrity. Potentially consistent with such collaboration, secreted phospholipases contribute to *Toxoplasma* egress [51] and *Plasmodium* cell traversal [52] and egress [53]. Regardless, much is yet to be learned about the molecular underpinnings of PFP function in the disrupting of biological membranes by apicomplexan parasites.

## Figures and Tables

**Table 1:** Proteins involved in apicomplexan cell traversal or egress.

Protein	Parasites	Type of PFP	*Toxoplasma* Stage/Function	*Plasmodium* Stage/Function
SPECT1	*Plasmodium* spp.	unknown	NA	sporozoite/cell traversal
CelTOS	Aconoidasida	α ^4^	NA	sporozoite and ookinete/cell traversal
PLP1/SPECT2	Apicomplexa ^1^	β	tachyzoite/egress	sporozoite/cell traversal, merozoite/egress
PLP2	Apicomplexa ^1^	β	sexual stages/unknown	male gametocyte/egress, merozoite/egress
PLP3//MAOP	Apicomplexa ^2^	β	NA	ookinete/cell traversal
PLP4	Apicomplexa ^3^	β	NA	ookinete/cell traversal
PLP5	Apicomplexa ^3^	β	NA	ookinete/cell traversal

^1^ Not present in *Cryptosporidium* or *Gregarina*. ^2^ Not present in *Cryptosporidium*, *Gregarina*, *Toxoplasma*, *Cyclospora*, or *Eimeria*. ^3^ Only present in Aconoidasida and *Cytauxzoon*. ^4^ Implied based on the crystal structure.

## References

[1] Davies A.P., Chalmers R.M. (2009). Cryptosporidiosis. BMJ.

[2] Rich S.M., Leendertz F.H., Xu G., LeBreton M., Djoko C.F., Aminake M.N., Takang E.E., Diffo J.L., Pike B.L., Rosenthal B.M. (2009). The Origin of Malignant Malaria. Proc. Natl. Acad. Sci. USA.

[3] Halonen S.K., Weiss L.M. (2013). Toxoplasmosis. Handb. Clin. Neurol..

[4] Foreyt W.J. (1990). Coccidiosis and Cryptosporidiosis in Sheep and Goats. Vet. Clin. N. Am. Food Anim. Pract..

[5] Anderson M., Barr B., Rowe J., Conrad P. (2012). Neosporosis in Dairy Cattle. Jpn. J. Vet. Res..

[6] Kim K., Weiss L.M. (2004). *Toxoplasma gondii*: The Model Apicomplexan. Int. J. Parasitol..

[7] Dal Peraro M., van der Goot F.G. (2016). Pore-Forming Toxins: Ancient, but Never really Out of Fashion. Nat. Rev. Microbiol..

[8] Dunstone M.A., Tweten R.K. (2012). Packing a Punch: The Mechanism of Pore Formation by Cholesterol Dependent Cytolysins and Membrane Attack complex/perforin-Like Proteins. Curr. Opin. Struct. Biol..

[9] Johnson T.K., Henstridge M.A., Warr C.G. (2017). MACPF/CDC Proteins in Development: Insights from Drosophila Torso-Like. Semin. Cell Dev. Biol..

[10] Ni T., Gilbert R.J.C. (2017). Repurposing a Pore: Highly Conserved Perforin-Like Proteins with Alternative Mechanisms. Philos. Trans. R. Soc. Lond. B. Biol. Sci..

[11] Mota M.M., Pradel G., Vanderberg J.P., Hafalla J.C., Frevert U., Nussenzweig R.S., Nussenzweig V., Rodriguez A. (2001). Migration of *Plasmodium* Sporozoites through Cells before Infection. Science.

[12] Amino R., Giovannini D., Thiberge S., Gueirard P., Boisson B., Dubremetz J.F., Prevost M.C., Ishino T., Yuda M., Menard R. (2008). Host Cell Traversal is Important for Progression of the Malaria Parasite through the Dermis to the Liver. Cell Host Microbe.

[13] Risco-Castillo V., Topcu S., Marinach C., Manzoni G., Bigorgne A.E., Briquet S., Baudin X., Lebrun M., Dubremetz J.F., Silvie O. (2015). Malaria Sporozoites Traverse Host Cells within Transient Vacuoles. Cell Host Microbe.

[14] Soldati D., Meissner M. (2004). *Toxoplasma* as a Novel System for Motility. Curr. Opin. Cell Biol..

[15] Kafsack B.F., Pena J.D., Coppens I., Ravindran S., Boothroyd J.C., Carruthers V.B. (2009). Rapid Membrane Disruption by a Perforin-Like Protein Facilitates Parasite Exit from Host Cells. Science.

[16] Acharya P., Garg M., Kumar P., Munjal A., Raja K.D. (2017). Host-Parasite Interactions in Human Malaria: Clinical Implications of Basic Research. Front. Microbiol..

[17] Robert-Gangneux F., Darde M.L. (2012). Epidemiology of and Diagnostic Strategies for Toxoplasmosis. Clin. Microbiol. Rev..

[18] Meis J.F., Verhave J.P., Jap P.H., Meuwissen J.H. (1983). An Ultrastructural Study on the Role of Kupffer Cells in the Process of Infection by *Plasmodium berghei* Sporozoites in Rats. Parasitology.

[19] Kafsack B.F.C., Carruthers V.B. (2010). Apicomplexan Perforin-Like Proteins. Commun. Integr. Biol..

[20] Ishino T., Yano K., Chinzei Y., Yuda M. (2004). Cell-Passage Activity is Required for the Malarial Parasite to Cross the Liver Sinusoidal Cell Layer. PLoS Biol..

[21] Yang A.S., O’Neill M.T., Jennison C., Lopaticki S., Allison C.C., Armistead J.S., Erickson S.M., Rogers K.L., Ellisdon A.M., Whisstock J.C. (2017). Cell Traversal Activity is Important for *Plasmodium falciparum* Liver Infection in Humanized Mice. Cell Rep..

[22] Hamaoka B.Y., Ghosh P. (2014). Structure of the Essential *Plasmodium* Host Cell Traversal Protein SPECT1. PLoS ONE.

[23] Holm L., Rosenstrom P. (2010). Dali Server: Conservation Mapping in 3D. Nucleic Acids Res..

[24] Brett T.J., Legendre-Guillemin V., McPherson P.S., Fremont D.H. (2006). Structural Definition of the F-Actin-Binding THATCH Domain from HIP1R. Nat. Struct. Mol. Biol..

[25] Hao W., Collier S.M., Moffett P., Chai J. (2013). Structural Basis for the Interaction between the Potato Virus X Resistance Protein (Rx) and its Cofactor Ran GTPase-Activating Protein 2 (RanGAP2). J. Biol. Chem..

[26] Zhao J., Beyrakhova K., Liu Y., Alvarez C.P., Bueler S.A., Xu L., Xu C., Boniecki M.T., Kanelis V., Luo Z.Q. (2017). Molecular Basis for the Binding and Modulation of V-ATPase by a Bacterial Effector Protein. PLoS Pathog..

[27] Yue P., Zhang Y., Mei K., Wang S., Lesigang J., Zhu Y., Dong G., Guo W. (2017). Sec3 Promotes the Initial Binary t-SNARE Complex Assembly and Membrane Fusion. Nat. Commun..

[28] Nishimura K., Han L., Bianchi E., Wright G.J., de Sanctis D., Jovine L. (2016). The Structure of Sperm Izumo1 Reveals Unexpected Similarities with Plasmodium Invasion Proteins. Curr. Biol..

[29] Ohto U., Ishida H., Krayukhina E., Uchiyama S., Inoue N., Shimizu T. (2016). Structure of IZUMO1-JUNO Reveals Sperm-Oocyte Recognition during Mammalian Fertilization. Nature.

[30] Kariu T., Ishino T., Yano K., Chinzei Y., Yuda M. (2006). CelTOS, a Novel Malarial Protein that Mediates Transmission to Mosquito and Vertebrate Hosts. Mol. Microbiol..

[31] Alves E., Salman A.M., Leoratti F., Lopez-Camacho C., Viveros-Sandoval M.E., Lall A., El-Turabi A., Bachmann M.F., Hill A.V., Janse C.J. (2017). Evaluation of Plasmodium Vivax Cell-Traversal Protein for Ookinetes and Sporozoites as a Preerythrocytic P. Vivax Vaccine. Clin. Vaccine Immunol..

[32] Espinosa D.A., Vega-Rodriguez J., Flores-Garcia Y., Noe A.R., Munoz C., Coleman R., Bruck T., Haney K., Stevens A., Retallack D. (2017). The *Plasmodium falciparum* Cell-Traversal Protein for Ookinetes and Sporozoites as a Candidate for Preerythrocytic and Transmission-Blocking Vaccines. Infect. Immun..

[33] Jimah J.R., Salinas N.D., Sala-Rabanal M., Jones N.G., Sibley L.D., Nichols C.G., Schlesinger P.H., Tolia N.H. (2016). Malaria Parasite CelTOS Targets the Inner Leaflet of Cell Membranes for Pore-Dependent Disruption. Elife.

[34] Tavares J., Amino R., Menard R. (2014). The Role of MACPF Proteins in the Biology of Malaria and Other Apicomplexan Parasites. Subcell. Biochem..

[35] Lukoyanova N., Hoogenboom B.W., Saibil H.R. (2016). The Membrane Attack Complex, Perforin and Cholesterol-Dependent Cytolysin Superfamily of Pore-Forming Proteins. J. Cell. Sci..

[36] Garg S., Sharma V., Ramu D., Singh S. (2015). In Silico Analysis of Calcium Binding Pocket of Perforin Like Protein 1: Insights into the Regulation of Pore Formation. Syst. Synth. Biol..

[37] Roiko M.S., Carruthers V.B. (2013). Functional Dissection of *Toxoplasma gondii* Perforin-Like Protein 1 Reveals a Dual Domain Mode of Membrane Binding for Cytolysis and Parasite Egress. J. Biol. Chem..

[38] Roiko M.S., Svezhova N., Carruthers V.B. (2014). Acidification Activates *Toxoplasma gondii* Motility and Egress by Enhancing Protein Secretion and Cytolytic Activity. PLoS Pathog..

[39] Garg S., Agarwal S., Kumar S., Shams Yazdani S., Chitnis C.E., Singh S. (2013). Calcium-Dependent Permeabilization of Erythrocytes by a Perforin-Like Protein during Egress of Malaria Parasites. Nat. Commun..

[40] Law R.H., Lukoyanova N., Voskoboinik I., Caradoc-Davies T.T., Baran K., Dunstone M.A., D’Angelo M.E., Orlova E.V., Coulibaly F., Verschoor S. (2010). The Structural Basis for Membrane Binding and Pore Formation by Lymphocyte Perforin. Nature.

[41] Yang A.S., Boddey J.A. (2017). Molecular Mechanisms of Host Cell Traversal by Malaria Sporozoites. Int. J. Parasitol..

[42] Ishino T., Chinzei Y., Yuda M. (2005). A *Plasmodium* Sporozoite Protein with a Membrane Attack Complex Domain is Required for Breaching the Liver Sinusoidal Cell Layer Prior to Hepatocyte Infection. Cell. Microbiol..

[43] Tavares J., Formaglio P., Thiberge S., Mordelet E., Van Rooijen N., Medvinsky A., Menard R., Amino R. (2013). Role of Host Cell Traversal by the Malaria Sporozoite during Liver Infection. J. Exp. Med..

[44] Pradel G., Frevert U. (2001). Malaria Sporozoites Actively Enter and Pass through Rat Kupffer Cells Prior to Hepatocyte Invasion. Hepatology.

[45] Deligianni E., Morgan R.N., Bertuccini L., Wirth C.C., Silmon de Monerri N.C., Spanos L., Blackman M.J., Louis C., Pradel G., Siden-Kiamos I. (2013). A Perforin-Like Protein Mediates Disruption of the Erythrocyte Membrane during Egress of *Plasmodium berghei* Male Gametocytes. Cell. Microbiol..

[46] Kadota K., Ishino T., Matsuyama T., Chinzei Y., Yuda M. (2004). Essential Role of Membrane-Attack Protein in Malarial Transmission to Mosquito Host. Proc. Natl. Acad. Sci. USA.

[47] Ecker A., Bushell E.S., Tewari R., Sinden R.E. (2008). Reverse Genetics Screen Identifies Six Proteins Important for Malaria Development in the Mosquito. Mol. Microbiol..

[48] Wade K.R., Tweten R.K. (2015). The Apicomplexan CDC/MACPF-Like Pore-Forming Proteins. Curr. Opin. Microbiol..

[49] Lopez J.A., Brennan A.J., Whisstock J.C., Voskoboinik I., Trapani J.A. (2012). Protecting a Serial Killer: Pathways for Perforin Trafficking and Self-Defense Ensure Sequential Target Cell Death. Trends Immunol..

[50] Brennan A.J., Chia J., Browne K.A., Ciccone A., Ellis S., Lopez J.A., Susanto O., Verschoor S., Yagita H., Whisstock J.C. (2011). Protection from Endogenous Perforin: Glycans and the C Terminus Regulate Exocytic Trafficking in Cytotoxic Lymphocytes. Immunity.

[51] Pszenny V., Ehrenman K., Romano J.D., Kennard A., Schultz A., Roos D.S., Grigg M.E., Carruthers V.B., Coppens I. (2016). A Lipolytic Lecithin:Cholesterol Acyltransferase Secreted by Toxoplasma Facilitates Parasite Replication and Egress. J. Biol. Chem..

[52] Bhanot P., Schauer K., Coppens I., Nussenzweig V. (2005). A Surface Phospholipase is Involved in the Migration of *Plasmodium* Sporozoites through Cells. J. Biol. Chem..

[53] Burda P.C., Roelli M.A., Schaffner M., Khan S.M., Janse C.J., Heussler V.T. (2015). A *Plasmodium* Phospholipase is Involved in Disruption of the Liver Stage Parasitophorous Vacuole Membrane. PLoS Pathog..

